# Key Disease Mechanisms Linked to Alzheimer’s Disease in the Entorhinal Cortex

**DOI:** 10.3390/ijms22083915

**Published:** 2021-04-10

**Authors:** Virginie Bottero, Dallen Powers, Ashna Yalamanchi, James P. Quinn, Judith A. Potashkin

**Affiliations:** 1 Center for Neurodegenerative Diseases and Therapeutics, Discipline of Cellular and Molecular Pharmacology, The Chicago Medical School, Rosalind Franklin University of Medicine and Science, North Chicago, IL 60064, USA; virginie.bottero@rosalindfranklin.edu (V.B.); dallen.powers@my.rfums.org (D.P.); ashna.yalamanchi@my.rfums.org (A.Y.); 2 Q Regulating Systems, LLC, Gurnee, IL 60031, USA; jim.quinn@qregulatingsystems.com

**Keywords:** Alzheimer’s disease, dementia, switch genes, entorhinal cortex

## Abstract

Alzheimer’s disease (AD) is a chronic, neurodegenerative brain disorder affecting millions of Americans that is expected to increase in incidence with the expanding aging population. Symptomatic AD patients show cognitive decline and often develop neuropsychiatric symptoms due to the accumulation of insoluble proteins that produce plaques and tangles seen in the brain at autopsy. Unexpectedly, some clinically normal individuals also show AD pathology in the brain at autopsy (asymptomatic AD, AsymAD). In this study, SWItchMiner software was used to identify key switch genes in the brain’s entorhinal cortex that lead to the development of AD or disease resilience. Seventy-two switch genes were identified that are differentially expressed in AD patients compared to healthy controls. These genes are involved in inflammation, platelet activation, and phospholipase D and estrogen signaling. Peroxisome proliferator-activated receptor γ (PPARG), zinc-finger transcription factor (YY1), sterol regulatory element-binding transcription factor 2 (SREBF2), and early growth response 1 (EGR1) were identified as transcription factors that potentially regulate switch genes in AD. Comparing AD patients to AsymAD individuals revealed 51 switch genes; PPARG as a potential regulator of these genes, and platelet activation and phospholipase D as critical signaling pathways. Chemical–protein interaction analysis revealed that valproic acid is a therapeutic agent that could prevent AD from progressing.

## 1. Introduction

Alzheimer’s disease (AD) is a chronic, progressive, neurodegenerative disease estimated to affect more than 24 million people worldwide [1]. This number is expected to double every 20 years, with a global prevalence of nearly 50 million by 2040 [1]. Despite the widespread prevalence of this disease, the underlying pathophysiology is poorly understood, and current medications and therapies are minimally effective. There is strong evidence to suggest that the proteins β-amyloid (Aβ) and tau are involved in the pathogenesis of Alzheimer’s disease. In AD patients, hyperphosphorylated tau and Aβ are produced in abundance. Tau deposits intracellularly, forming bundles that interfere with appropriate intracellular transport [2]. Aβ aggregation leads to inter-neuronal plaques that interfere with proper signaling and communication, including the modulation of neurotropins, neurotransmitter receptors, lipid metabolism, and innate immunity, that interferes with brain function. The toxic environment induced by Aβ includes mitochondrial dysfunction, reactive oxygen species, and inflammation [3]. Risk factors associated with AD include genetic predisposition; advanced age; and environmental changes related to lifestyle factors, such as poor diet and sedentary behaviors. AD risk is also associated with lipid metabolism, inflammation, cerebrovascular accidents, and head trauma [4].

Current diagnostic practices require both cognitive and pathologic findings. It is generally believed that the pathological changes start to occur years before the onset of any symptoms. Clinical symptoms include progressive worsening of memory impacting daily activities, leading to dependency and disability [5]. Furthermore, pathological findings include accumulation of extracellular Aβ and hyperphosphorylated tau filaments in the brain, forming insoluble plaques and tangles, respectively [6]. The availability of biomarkers of Aβ and tau pathology and MRI measures of atrophy have enabled earlier identification of disease onset and progression [7]. Guidelines of the National Institute on Aging and the Alzheimer’s Association (NIA-AA) provide diagnostic criteria for AD based on biomarkers obtained in vivo or post-mortem examination [8]. Among AD individuals, some patients will present cognitive impairment as well as AD pathology (AD), whereas individuals with intact cognition but neuropathological findings consistent with AD are referred to as asymptomatic Alzheimer’s disease individuals (AsymAD) [9].

Up to 20–30% of the aging population with intact cognition have Aβ deposition, with these individuals at higher risk of progressing to AD than those without Aβ [10]. AsymAD individuals may be distinguished from normal aging based on neuropathology, brain imaging, and cerebrospinal fluid biomarkers [11]. As many as 30–50% of older individuals who come to autopsy as clinically normal have AD pathology present in the brain [9]. It is not clear whether these individuals would become AD patients if they had lived longer or if they may be resilient to AD.

The entorhinal cortex is a critical brain region in which AD related neurodegeneration appears [12]. Functional magnetic resonance imaging specifically identified the lateral entorhinal cortex as the first region affected by AD before it spreads to other brain regions [13]. These early changes in the entorhinal cortex allow it to be used as a strong predictor of AD in the prodromal phase [14]. Since the entorhinal cortex plays an essential role in memory formation and learning and is the main relay pathway between the hippocampus and neocortex, its dysfunction can lead to mild cognitive impairment and dementia [15]. Recently, gene expression profiling of laser-capture entorhinal cortex neurons from post-mortem AD and control brains identified specific changes that initiated the cascade of events leading to AD pathology [16].

The key inciting factors responsible for the development of AD remain unknown, but using SWItchMiner software (SWIM), gene expression studies of post-mortem brain tissue may reveal pathways implicated in AD’s development. SWIM allows for the combination of gene expression networks with topological properties of correlation networks to reveal important hubs located amongst the networks that may have important features [17]. Some of these hubs, denoted as switch genes, are characterized by extensive connections throughout the network and are assigned a critical topographical role, indicating their significance in pathologies [17,18]. Previously, switch genes in human cancer networks and other prominent disease networks were identified [17]. In our earlier studies, the application of SWIM allowed us to identify several switch genes associated with dementia [19,20]. This study applied the SWIM algorithm to the gene expression dataset GSE118553 from AD and AsymAD patients’ entorhinal cortex. We further analyzed these switch genes to determine the dysregulated pathways and potential regulatory transcription factors and miRNAs that may be important for the development of AD or for disease resilience.

## 2. Results

### 2.1. Identification of Switch Genes for Entorhinal Cortex between AD Versus Healthy or AsymAD

**Figure 1:**
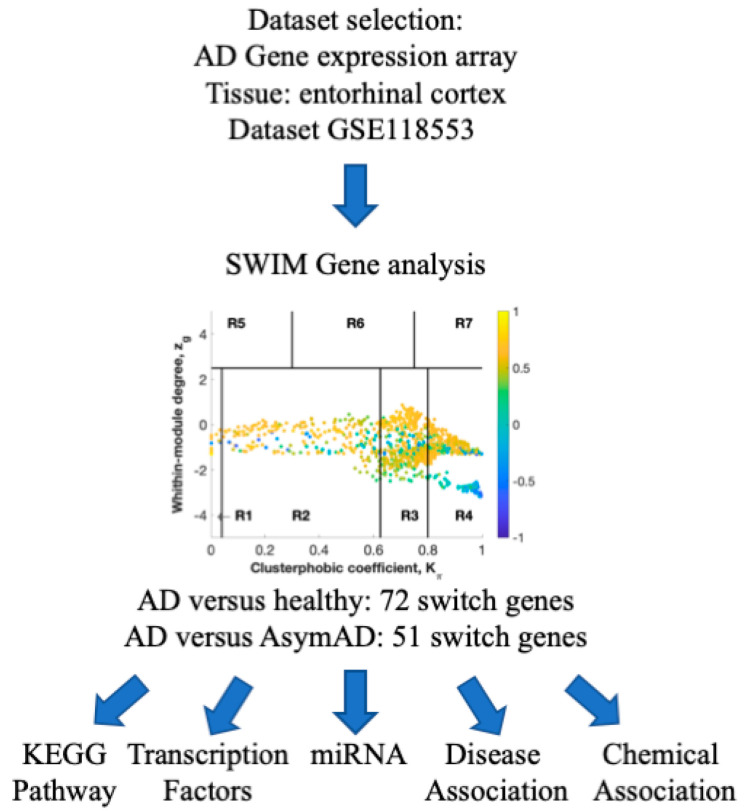
Flowchart of the study. SWIM analysis was performed to identify switch genes, which were further analyzed for functional pathways, regulatory transcription factors and miRNAs, and disease and chemical associations. AD: Alzheimer’s disease. AsymAD: asymptomatic AD. SWIM: SWItchMiner software.

To identify genes that may control AD’s development in the entorhinal cortex, we used SWIM software to analyze the dataset GSE118553 [11]. The overall strategy of the study is depicted in Figure 1.

**Figure 2:**
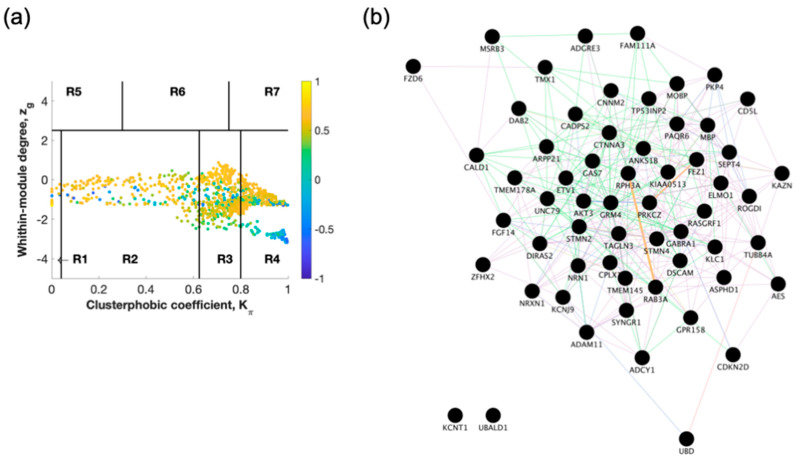
Identification of Alzheimer’s disease (AD) versus healthy switch genes. (**a**) Heat cartography maps of nodes of the AD/healthy correlation networks from the GSE118553 dataset. Dots correspond to nodes in the AD correlation networks and are distributed across seven regions (R1 to R7) according to their clusterphobic coefficient *Kπ* (*x*-axis) and according to their within-module degree *Zg* (*y*-axis). Region R4 represents the switch with nodes. (**b**) Network analysis. Gene network analysis was performed using GeneMANIA in Cytoscape v3.8.0. Input genes are shown in black circles. Purple, blue, and pink lines represent co-expression, co-localization, and physical interactions, respectively.

**Figure 3:**
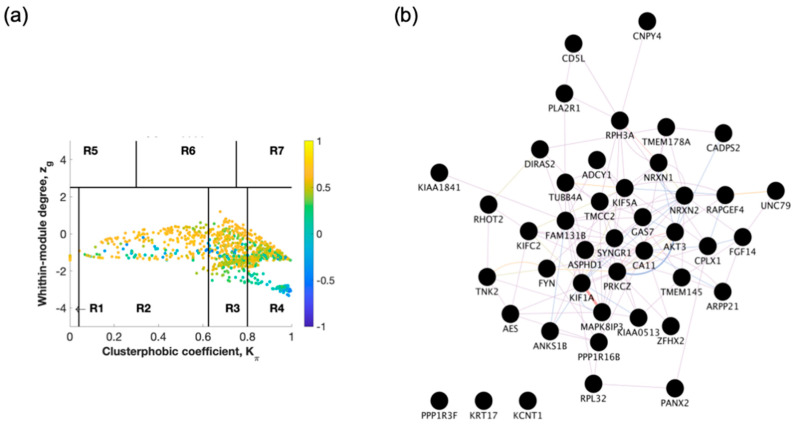
Identification of AD versus asymptomatic AD (AsymAD) switch genes. (**a**) Heat cartography maps of nodes of the AD/AsymAD correlation networks from the GSE118553 dataset as previously mentioned. (**b**) Network analysis. Gene network analysis was performed using GeneMANIA in Cytoscape v3.8.0. Input genes are shown in black circles. Purple, blue, and pink lines represent co-expression, co-localization, and physical interactions, respectively.

The raw gene expression dataset (GSE118553) from entorhinal cortex brain tissue from demented individuals was imported into SWIM. The analysis was performed comparing AD to healthy control (Figure 2 and Figure S1, Table S1) and AD to AsymAD (Figure 3 and Figure S2, Table S1). In the first step, genes were retained (red bars) or eliminated (grey bars) according to the selected fold-change threshold of 1.5 (Figures S1a and S2a). In the second step, the average Pearson correlation coefficient allowed the identification of correlation communities (Figure 2a and Figure 3a). Yellow nodes are party and date hubs, which are positively correlated in expression with their interaction partners. Blue nodes are the fight club hubs with an average negative correlation in expression with their interaction partners. Blue nodes falling in the region R4 are the switch genes characterized by low within-module degree *Zg* and high clusterphobic coefficient *K*π values and are connected mainly outside their module. In the third step, the expression profiles of switch genes are clustered according to rows (switch genes) and columns (samples) of the switch gene expression data (biclustering) (Figures S1b and S2b). In general, the switch genes are downregulated in AD. In the final step, the robustness of the analysis is determined (Figures S1c and S2c). The results show that the fight club hubs are readily discernable from the date and party hubs.

SWIM analysis identified 72 switch genes comparing AD and healthy individuals, including 59 protein-coding genes, 2 non-coding RNAs (HAR1A and RNU4ATAC), and 11 chromosomal loci (Table S1). Fifty-one switch genes were discovered comparing AD and AsymAD corresponding to 43 protein-coding genes, 2 RNAs (MEG3 lncRNA and SCARNA11), and 6 chromosomal loci (Table S1). The switch genes were imported in Cytoscape v3.8.0 to determine the co-expression, co-localization, and physical interactions between the genes (Figure 2b and Figure 3b). Twenty-five genes were shared between the AD/healthy, and AD/AsymAD analysis, and 26 genes were unique to AD/AsymAD (Table S1).

### 2.2. Pathway Enrichment Analysis

**Figure 4:**
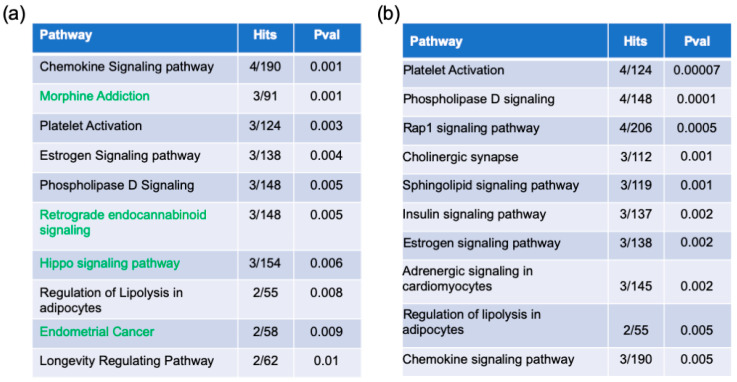
Top 10 pathways. (**a**) Top 10 pathways identified from the AD/healthy switch genes. (**b**) Top 10 pathways identified from AD/AsymAD switch genes. The pathways shared between both analyses are indicated in black. The pathways shown in green are unique for each analysis. Pval: *p*-value.

The switch genes’ biological and functional roles were determined by pathway analysis using the Kyoto Encyclopedia of Genes and Genome (KEGG) database in NetworkAnalyst. Thirty-eight pathways were identified from the AD/healthy switch genes (Figure 4a, Table S2). Interestingly, many of the pathways identified are associated with infection and inflammation. Similarly, 29 pathways were identified from the AD/AsymAD switch genes (Figure 4b, Table S2). Finally, 20 pathways were shared between both analyses (Table S2). Nine pathways were unique to AD/AsymAD (cAMP signaling, Fc epsilon RI signaling, endocrine resistance, T cell receptor signaling, Chagas disease, osteoclast differentiation, cell adhesion molecules, cGMP-PKG signaling, tuberculosis, and axon guidance).

### 2.3. Gene–Transcription Factor Interaction Analysis

**Figure 5:**
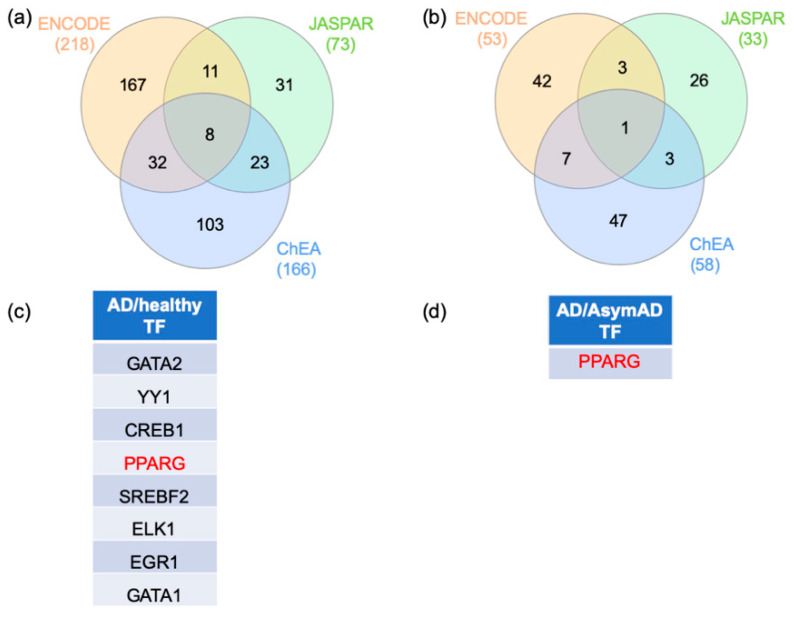
Transcription factors analysis. Transcription factors analysis for the entorhinal cortex from AD/control and AD/AsymAD patients. The gene–transcription factor interaction network was performed with ENCODE, ChEA, and JASPAR. Venn diagram analysis was performed to identify the transcription factors identified by the three methods for AD/healthy (**a**,**c**) and AD/AsymAD (**b**,**d**). AD: Alzheimer’s disease. AsymAD: asymptomatic AD. TF: Transcription factor.

In order to identify the central regulators of the switch genes, the gene–transcription factor interactomes were performed on NetworkAnalyst using three different databases (ENCODE, ChEA, and JASPAR) (Table S3). The AD/healthy switch genes’ analysis identified 218, 73, and 166 transcription factors from ENCODE, JASPAR, and ChEA, respectively. Eight transcription factors were shared amongst the three analyses (GATA1, GATA2, YY1, CREB1, PPARG, SREBF2, ELK1, and EGR1) (Figure 5a,c). The AD/AsymAD switch genes analysis identified 53, 33, 58 transcription factors from ENCODE, JASPAR, and ChEA, respectively. Only PPARG was shared by the three analyses (Figure 5b,d). Interestingly, PPARG was also shared between AD/healthy and AD/AsymAD analyses.

### 2.4. Gene–miRNA Interaction Analysis

**Figure 6:**
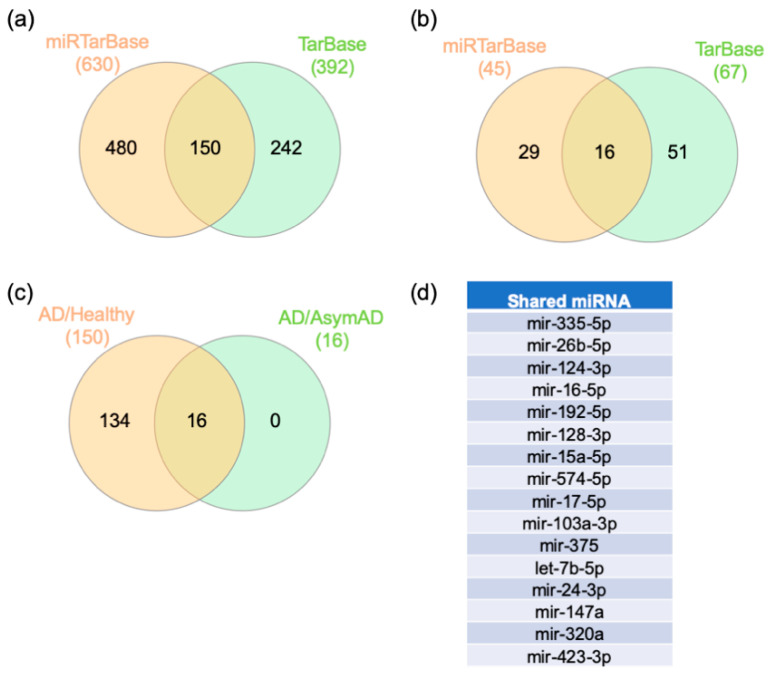
Analysis of miRNAs. Analysis of miRNAs from AD/control and AD/AsymAD switch genes. The gene–transcription factor interaction network was performed with miRTarBase and TarBase. Venn diagram analysis was performed to identify the miRNAs for AD/healthy (**a**) and AD/AsymAD analysis (**b**) shared between the databases. A Venn diagram determined the miRNA shared between both analyses (**c**,**d**). AD: Alzheimer’s disease. AsymAD: asymptomatic AD.

To further study the regulation of the switch genes’ expression, a gene–miRNA interaction network analysis was performed in NetworkAnalyst. Comprehensive experimentally validated miRNA–gene interaction data were collected from TarBase v.8.0 and miRTarBase v.8.0. One hundred fifty miRNAs and 16 miRNAs were shared between the databases that potentially regulate the AD/healthy and AD/AsymAD switch genes, respectively (Figure 6a,b, Table S4). Interestingly, all 16 miRNAs identified in the AD/AsymAD analysis were also identified in the AD/healthy analysis (Figure 6c,d).

### 2.5. Gene–Disease Association Analysis

**Figure 7:**
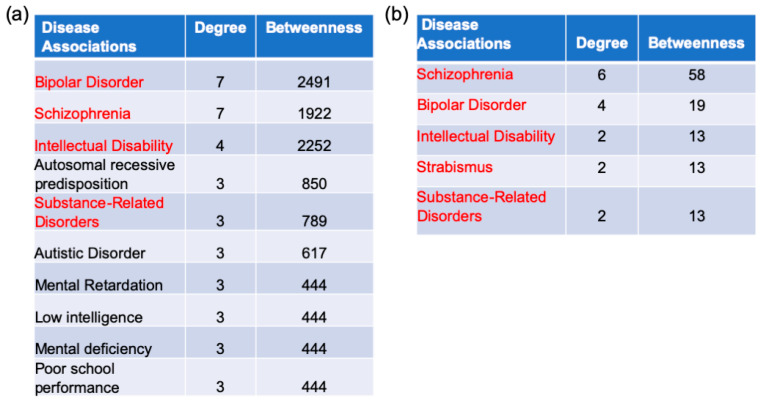
Disease association analysis. The list of top 10 disease associations, ranked by degree and betweenness, obtained from AD/healthy and AD/AsymAD switch genes is shown in (**a**,**b**). The associated diseases shared between both analyses are indicated in red.

To further understand pathologies related to AD, a gene–disease network analysis was performed in NetworkAnalyst. The literature-curated gene–disease relationships were collected from the DisGeNET database. The AD/healthy switch genes analysis identified 146 disease pathways (Figure 7a, Table S5). Of these 146, the top 10 pathways were bipolar disorder, schizophrenia, intellectual disability, autosomal recessive predisposition, substance-related disorders, autistic disorder, mental retardation, low intelligence, mental deficiency, and poor school performance. In contrast, the analysis from AD/AsymAD switch genes identified only five diseases: schizophrenia, bipolar disorder, intellectual disability, strabismus, and substance-related disorders (Figure 7b, Table S5).

### 2.6. Protein–Chemical Interaction Analysis

**Figure 8:**
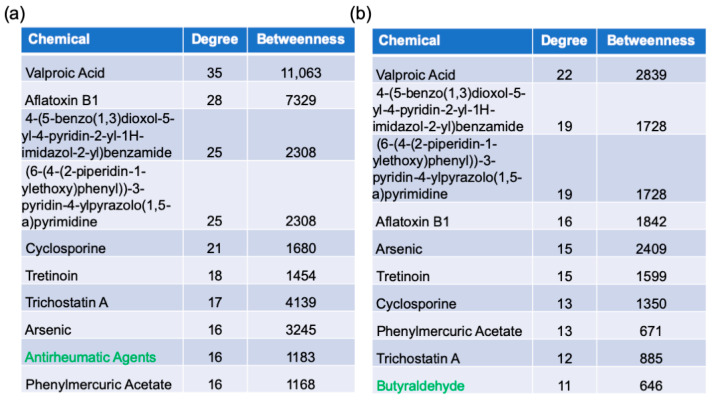
Protein–chemical interaction analysis. NetworkAnalyst was used to identify potential therapeutic reagents. The top 10 chemicals identified from the AD/healthy and AD/AsymAD switch genes are shown in (**a**,**b**), respectively. The chemicals shown in green are unique for each analysis.

Drugs that are potentially useful for treating AD were determined by protein–chemical interaction network analysis in NetworkAnalyst. The data are based on data from the Comparative Toxicogenomics Database. The analysis identified 324 and 233 chemicals interacting from the AD/healthy and AD/AsymAD switch genes analysis, respectively (Table S6). The top 10 chemicals are listed in Figure 8. Interestingly, 173 chemicals were shared between the analyses, including 9 of the top 10 chemicals identified.

## 3. Discussion

### 3.1. Genes

Many switch genes shared between AsymAD and AD were identified by SWIM in this study, suggesting that both groups share dysregulated gene expression that may lead to pathology. The switch gene *AKT3* has important roles in both insulin sensitivity and neuroinflammation, which have been implicated in the pathogenesis of AD [21]. One potential role is through the effects of miR-485-3p, a miRNA targeting *AKT3* and a potential therapeutic biomarker for AD. Increased serum levels of miR-485-3p and subsequent knockdown of *AKT3* expression correlated with an increased inflammatory response in AD patients and a significant decrease in neuronal viability measured in vitro by MTT and cell apoptosis analyses [22]. Furthermore, appropriate regulation of *AKT3* gene function has a positive effect on insulin signaling. Increased administration of insulin and GLP-1 agonists in mice afflicted with AD modulated *AKT3* gene expression and other mediators of insulin signaling [23]. The increased insulin sensitivity leads to a decreased Aβ level and suggests that further investigation into insulin combination therapy is warranted [23]. Properly regulated *AKT3* gene expression may be neuroprotective in other inflammatory neurological disorders, such as amyotrophic lateral sclerosis (ALS) and Parkinson’s disease (PD) [24,25].

Another switch gene identified in our study is *ANKS1B*, coding for the protein AIDA-1. *ANKS1B* may be important in late-stage AD pathogenesis [26]. Overexpression of Aβ is associated with the disparate intracellular distribution of AIDA-1 [27]. AIDA-1 binds intracellular Aβ domains and may play a role in regulating Aβ plaque build-up in AD [28], suggesting it may be a therapeutic target for AD [27]. Dysregulation of AIDA-1 and other synaptonuclear messengers is related to neuron synaptic failure seen in AD and other neurodegenerative pathologies [29].

The switch gene *CLPX1*, identified in our analysis, is involved in the production of complexins, which are presynaptic proteins that significantly affect the release of neurotransmitters [30]. Inappropriate expression of these proteins has been implicated in neurodegenerative disorders, such as AD and PD. *CLPX1* knockout mice showed significant behavioral impairment compared to controls, and dysregulation of the *CLPX1* gene has been implicated in the life-altering behavioral and cognitive impairment seen in AD. Further evidence of this trend was provided by a recent study, which showed that appropriate expression of CLPX1 promoted cognitive resilience in elderly patients and significantly diminished the risk of development of AD or any similar symptomatology [31]. CLPX1 has also been implicated in the dysregulation of frontotemporal SNARE proteins and subsequent development of AD pathology [32]. Specifically, increased SNARE proteins and an increased CLPX1/CLPX2 ratio were found to be neuroprotective in elderly patients [32].

The accumulation of hyperphosphorylated tau has been heavily implicated in AD’s pathogenesis. The protein encoded by the switch gene *GAS7* that we identified inhibits the production of phosphorylated tau by binding to its C-terminal domain and preventing conversion into fibrils and blocking aggregation, thus potentially playing a neuroprotective role in AD [33]. Both low and high levels of GAS7 in neurons have been implicated in the pathogenesis of AD progression. Elevated levels of GAS7 interfere with neuron microtubule transport proteins, such as kinesin, which may disrupt the homeostasis of healthy tau in the central nervous system (CNS) [34]. These findings suggest that dysregulated expression of GAS7 in the CNS may contribute to increased susceptibility and risk for the development of AD [35].

The *FGF14* switch gene revealed in our analysis has emerged as a risk factor for developing neurological brain disease due to its importance in controlling voltage-gated sodium channels in initial axon segments [36]. The absence of the *FGF14* gene in knockout mice led to aberrant sodium channel signaling, as well as dysfunction and behaviors associated with schizophrenia and other neurological disorders [37]. Furthermore, animal models have demonstrated that Aβ pathology can be ameliorated through the use of PPAR-γ agonists, such as the thiazolidinedione class of medications, which are commonly prescribed diabetes medications [38]. Administration of these medications results in FGF14 phosphorylation on the S226 residue and modulates sodium channel signaling in the dentate gyrus and other insulin-sensitive pathways [38]. This connection suggests the important role FGF14 may play as a PPAR-γ target in controlling neuronal dysfunction and memory-loss seen in early AD [38].

In addition, NRXN1 was identified as a switch gene in this study. Neurexins (NRXN) serve an important function in neuron synapse connection and signal transmission by promoting cellular adhesion. Neurexins have been implicated in the development of many different cognitive diseases, from autism to schizophrenia [39]. Fluctuations in NRXN1 levels and other neurexins are implicated in disrupting the balance of excitatory and inhibitory signals at synapses, resulting in damage and cognitive impairment seen early in AD [40]. NRXN1 has been found to interact with the Aβ plaques in AD leading to synaptic transmission impairment [41]. Presenilins, proteases involved in Aβ formation, proteolytically process neurexins, and the dysfunction of this pathway may be associated with AD [42]. In addition, inhibition of specific presenilins by pharmacological or other means resulted in the accumulation of neurexin fragments in neuron synapses found in the hippocampus in rat models, which is heavily damaged in the early stages of AD [43].

### 3.2. Pathways

Chemokine signaling was identified from the AD versus healthy analysis, suggesting that neuroinflammation plays a significant role in the AD development and pathogenesis of AD. Upregulation of chemokine signaling has been shown to influence kinases’ activity that leads to phosphorylation of tau [44]. Activation of microglial cells is an essential aspect of beneficial neuroinflammation; however, dysregulation of this process can severely alter the environment in which neurons and glial cells grow and develop. Excessive microglial activity due to overactive chemokine signaling in the CNS has been shown to increase the degeneration of neuroprotective substances, such as retinoic acid [45]. Increased prostaglandin activity decreases microglial activity, leading to the impaired clearing of misfolded proteins, improper regulation of inflammation, and impaired CNS tissue healing [46]. Furthermore, the Aβ plaques found in AD patients activate the nuclear factor kappa-light-chain-enhancer (NF-κB), upregulating the transcription of cytokines and chemokines involved in inflammation [47]. These chemokines induce oxidative stress and may contribute to the excessive neuroinflammation found in patients with AD [47]. The chemokine CXCL10, which interacts with receptor CXCR3, is highly elevated in AD patients [48]. Decreased CXCR3 signaling decreases Aβ burden load by enhancing microglial clearance of misfolded proteins, indicating that increased CXCR3 signaling may be related to AD pathology [47].

The platelet activation pathway was the most significant pathway identified from the AD versus AsymAD analysis. Ischemic attacks and compromised blood supply to the CNS are heavily connected to AD [49,50]. Mean platelet volume and platelet distribution width are abnormal in AD patients and may indicate platelet dysfunction [51,52]. These platelet characteristics may be useful early diagnostic biomarkers of AD [53]. Activation of platelets generates precursor proteins that ultimately can result in the deposition of Aβ peptides seen in AD, and a correlation between platelet activation and platelet count with Aβ levels has been suggested [54]. In addition to cleaving APP to form Aβ, platelets themselves can release Aβ peptide. Platelets are the primary source of Aβ in the blood, representing 90% of Aβ peptide in the blood [54]. Both APP and Aβ peptides can be released upon platelet degranulation [55]. Moreover, if activated platelets adhere to endothelial cells located in brain vessels, enzymes within these vessels can cleave APP and form Aβ. These Aβ peptides are very similar to those found in the senile plaques of AD patients. Two isoforms of the APP protein were detected by Western blotting in intact platelets (120–130 kDa and 110 kDa). The quantification of these isoform ratios is an indicator of AD progression [56,57]. Thus, measuring APP isoforms can be used as a helpful measure in diagnosing AD at its early stages, monitoring disease progression, and evaluating patients’ response to therapeutic interventions [55].

Phospholipases are universal enzymes that catalyze the conversion of their principal substrate phosphatidylcholine to phosphatidic acid. Phospholipase D (PLD) signaling was identified in this study as a pathway related to AD/healthy and AD/AsymAD switch genes (Figure 4). PLD is related to many processes affecting many vital cell functions, such as cellular metabolism, exocytosis, endocytosis, cytoskeletal reorganization, and, consequently, implicated in numerous diseases, including AD [58,59]. In European and African populations with late-onset AD, 9% have been associated with multiple rare *PLD3* polymorphisms [60]. PLD3 loss-of-function increases pathogenic Aβ peptide secretion. Moreover, PLD1 has protective effects in AD, acting as a negative regulator of Aβ formation in cell culture studies. Increased PLD signaling increases the adverse effects of Aβ plaques in AD patients and may increase Aβ load [61]. Elevated PLD1, found in AD patients’ hippocampus, has been proposed to cause synaptic dysfunction and subsequent memory disruption seen in AD [62]. Conversely, suppressing inappropriate PLD signaling in AD brains increases synaptic resilience, potentially slowing cognitive decline and providing therapeutic benefit [63]. *PLD2* ablation was shown to ameliorate memory deficits and offer synaptic protection in AD brains, despite the numerous tau and Aβ tangles and plaques [64].

Other shared pathways in the AD/healthy and AD/AsymAD analyses are related to the hormone insulin and insulin homeostasis. Several researchers have labeled AD as “type 3 diabetes” due to the high prevalence of the disease amongst those who suffer from diabetes mellitus and insulin resistance [21]. Brain insulin resistance has been demonstrated in early AD through decreased IR, IRS-1, and PI3K signaling in mildly symptomatic as well as severely symptomatic patients [65]. There is increasing evidence that the Aβ plaques and phosphorylated tau tangles seen in AD may affect pancreatic beta cells and the CNS, potentially leading to dysregulation of insulin homeostasis and subsequent disruption in glucose metabolism of the brain [66]. This phenomenon could explain the shift towards ketone metabolism use seen in AD brains and suggests that AD could be considered a neuro-metabolic disorder [66]. Insulin resistance is linked to the neuroinflammation seen in AD, and it downregulates PPARD, a hormone receptor essential to the development of AD [67]. PPARD functions as an anti-inflammatory agent in the CNS, and downregulation of this hormone receptor is implicated in the inflammatory processes seen in AD [67]. Insulin’s effect on oxidative stress has also been hypothesized to contribute to the inflammatory response seen in AD related to insulin resistance. Increased thioredoxin-interacting protein, which is thought to be an amplifier of oxidative stress and inflammasome activation and may mediate CNS insulin resistance, has been observed in AD patients with insulin resistance [68]. These are only a few examples of insulin’s complex effect on the CNS and its importance in AD’s pathogenesis. A further understanding of this hormone and its role in the development of AD could potentially lead to new therapeutics and treatment strategies for a disease that is still poorly understood [69].

### 3.3. Transcription Factors

Many transcription factors are involved in regulating the switch genes localized to the entorhinal cortex in AD patients. PPARG was identified here as a putative transcription factor that may regulate switch genes in AD and AsymAD. PPARG regulates the function of peroxisomes, cellular organelles involved in fatty-acid oxidation, and other metabolic processes. Elevated PPARG activity is related to a higher incidence of obesity and impaired insulin signaling, two contributing factors to AD [70,71]. In addition, individuals with a specific single nucleotide polymorphism (rs1805192) in *PPARG* were at higher risk to develop AD [72]. Finally, a significant association between the Pro12Ala genotype of *PPARG* and an increased rate of cognitive decline was observed among older black males [73]. PPARG has long been suggested as a molecular target for both gene therapy and pharmacological treatments due to the attenuation of AD pathology [74].

Another transcription factor regulating the transition from healthy or AsymAD to AD that we identified is YY1. YY1 mediates many genes necessary for neuronal survival and, thus, when dysregulated, leads to neuronal death and neurodegeneration [75]. Increased function of the multifunctional zinc-YY1 increases activity of the Aβ precursor protein-cleaving enzyme 1 (BACE1) involved in the regulation of Aβ degradation [76]. Excessive expression of YY1 methylates the *Fuz* gene promoter and decreases transcription, thus influencing planar cell polarity and subsequent cell stability [77]. Increased *Fuz* transcript levels were found in patients with AD pathology, indicating that YY1 gene modification may play a role in Fuz-related neuron apoptosis and resultant neurodegeneration [77]. YY1 regulation also modulates the activity of the gene *APH1A*, which ultimately transcribes a γ secretase involved in the cleavage of internal proteins, such as Aβ, in the AD brain [78].

Sterol regulatory element-binding protein 2 (SREBF2) is another transcription factor identified in this study that is involved in AD pathogenesis. SREBF2 is a ubiquitously expressed TF involved in the regulation of lipid metabolism and homeostasis [79]. Increased expression of SREBF2 levels measured in the frontal cortex is positively correlated with tau and Aβ levels in AD brains but inversely correlated with time of death [80]. Increased tau levels are also correlated with disruption of SREBF2 signaling, leading to further neurodegeneration [81]. Overexpression of SREBF2 exacerbates Aβ accumulation in neuronal cells and increases synaptotoxicity and memory deficits [82]. High levels of brain cholesterol that may result from defective SREBF2 signaling enhance autophagosome formation but impair the fusion of endosomes with lysosomes [83]. This impaired fusion leads to insufficient clearance of Aβ plaques and aggravates oxidative stress placed on neuronal cells [83].

Finally, EGR1, a transcription factor involved in cell differentiation and mitogenesis that we identified, has been suggested as a significant regulator of neuronal plasticity and in playing a role in both neurological and psychiatric disorders, as well as neurodegeneration [84]. EGR1′s relation to cell differentiation suggests a potential link between aging and AD interactions [85]. Acetylcholine is the primary neurotransmitter depleted in AD. EGR1 levels modulate acetylcholinesterase mRNA and protein, suggesting EGR1 may significantly contribute to the changes in acetylcholine signaling seen in AD [86]. Furthermore, EGR1, through its action on miRNA-132, was found to modulate the nucleus basalis of Meynert, an area rich in acetylcholine [87].

### 3.4. miRNA

We also identified many miRNAs that may regulate the switch genes identified in this study. One of these miRNAs, mirR-26b-5p, may be a biomarker for the premortem diagnosis of AD [88,89]. The downregulation of mirR-26b-5p targets was enriched in components needed to recognize the RNA polymerase II promoter, p53 signaling, and miRNAs in cancer pathways [89]. Increased Aβ plaque load in AD patients has also been connected with increased activity of the miR-26b-5p [90].

Downregulation of miR-124-3p, a putative switch gene regulator from our analysis, was found to lead to a large increase in hyperphosphorylated tau [91]. Increased miR-124-3p expression resulted in higher activation of caveolin-1, phosphoinositide 3-kinase (PI3K), phospho-AKT (AKT), and phospho-glycogen synthase-3 beta (GSK) [91]. Regulation of the caveolin-1/PI3K/AKT/GSK pathway was related to the inverse effect of miR-124-3p on tau. miR-124-3p is important in regulating calpain activity, where decreased levels of the miRNA lead to increased calpain activity and inappropriate hyperphosphorylation of tau [92]. Injection of miR-124-3p into AD brains decreased the amount of hyperphosphorylated tau, elucidating its vital importance in developing AD pathology [92]. In addition, increased expression of miR-124-3p in the microglia of an AD brain was found to reduce neurodegeneration and improve cognitive ability by targeting transcription factors related to ApoE, which promotes Aβ plaque breakdown [93]. Further research is needed to discover the important neuroprotective effects of this miRNA in AD.

In addition, our study revealed miR-16-5p as a potential regulator of the switch genes. Altered miR-16-5p was extracted from young-onset AD patients’ cerebrospinal fluid, suggesting an important role in the pathogenesis of AD [94]. Increased miR-16-5p co-localized with heavy Aβ plaque regions in AD brains, suggesting an important relationship between the miRNA and plaque deposition in AD pathology [95]. Altered miR-16-5p expression is related to the increased incidence of cancer and AD in the elderly, although the mechanism is complex [96].

### 3.5. Disease Association

Our network analysis identified many diseases related to AD switch genes, including neurological and psychiatric disorders. Bipolar disorder, a psychiatric disorder characterized by bouts of depression and periods of elevated moods, was associated with AD switch genes [97]. Brains of individuals with bipolar disorder have a high level of inflammation and increased cytokine levels, known instigators in neurodegeneration, and potential links between bipolar disorder and AD [98]. Deficits in the electron transport chain complex proteins I and IV, NADH dehydrogenase and c-oxidase, respectively, were dysregulated in both AD and bipolar disorder, along with a host of other neuropsychiatric complications. Examination of postmortem brains of individuals afflicted with bipolar disorder revealed tangles composed of hyperphosphorylated tau and subsequent neurodegeneration similar to AD pathology [99]. Administration of the commonly used drug for bipolar disorder lithium decreased cis phosphorylated tau levels and reduced subsequent neurodegeneration, suggesting a potential therapeutic option for bipolar disorder and AD [99]. However, lithium administration disrupts neuronal iron homeostasis through tau suppression, indicating further research is required [100].

Schizophrenia, a severe psychiatric disorder related to reality distortion, is potentially linked to AD. Degeneration of both the hippocampus and amygdala, known early targets in AD, was also found to be significant in schizophrenia, with varying degrees of similarity to AD patients depending on the subject [101]. Increased Aβ plaques were found in schizophrenic patients, suggesting similar pathophysiology to AD, although the levels of Aβ were still higher in AD subjects [102]. Dysregulation of calcium signaling in the CNS, specifically related to the ascending arousal system, has been implicated in both schizophrenia and AD pathology, with medications targeting these pathways providing some relief [103]. Aberrant Wnt signaling, crucial for appropriate early development of the CNS, correlates with increased Aβ neurotoxicity in AD [104]. Core components of Wnt signaling were also disrupted in schizophrenia, suggesting a similar etiology between schizophrenia and AD [104].

### 3.6. Chemicals

Many chemicals and commonly used therapeutic drugs that potentially interact with the switch genes or their encoded proteins were identified in this study. Valproic acid, for example, is a widely used medication for seizures; migraines; and other neuropsychiatric disorders, such as bipolar disorders [105]. As noted above, bipolar disorder was found to be associated with AD in our study. Interestingly, the risk of dementia is increased by a history of bipolar disorder [106]. Furthermore, the histone deacetylation action of valproic acid has been shown to downregulate amyloid precursor protein [107]. The use of valproic acid also attenuates Aβ load in AD pathology by inhibiting mitochondrial-mediated apoptosis, suggesting a potential important neuroprotective role of valproic acid in the treatment of AD [108]. Furthermore, the use of valproic acid in AD mouse models reduced brain inflammation and helped ameliorate memory deficits [109]. Recently, the combination of valproic acid with estrogen showed some therapeutic benefit on ovariectomized mice with AD [110]. Conversely, excessive, chronic use of valproic acid contributes to atrophy and degeneration of the hippocampus and reduced brain volume [111]. Further research is needed to delineate the potential role of valproic acid in the pharmacological treatment of AD. Interestingly, an earlier study that identified switch genes in the frontal cortex of AD, vascular dementia, and frontotemporal dementia patients also revealed valproic acid as a potential therapeutic target [20].

Antirheumatic agents were also identified in our chemical study. Rheumatoid arthritis is a chronic inflammatory disease primarily affecting the joints. Patients who have rheumatoid arthritis are often prescribed an anti-inflammatory drug regimen that may include nonsteroidal anti-inflammatory drugs (NSAIDs); corticosteroids; or disease-modifying antirheumatic drugs, such as methotrexate or hydroxychloroquine [112]. People with rheumatoid arthritis are at a slightly higher risk of developing AD [113]. However, pharmacologic treatment of rheumatoid arthritis has a neuroprotective effect and reduces AD risk [114]. Neuro-inflammation is heavily involved in AD’s pathogenesis, and specific biomarkers, such as inflammatory cytokines, are often indicative of progression [115]. Many mechanisms are proposed for the neuroprotective effects of antirheumatic agents in AD. NSAIDs and corticosteroids may attenuate the activation of complement by Aβ plaques and reduce subsequent neuron inflammation and destruction [114]. The anti-inflammatory agent aspirin reduces Aβ plaque pathology in AD mouse models through its action on the PPARG transcription factor [116]. Intrathecal steroidal medications, such as corticosteroids, may reduce potent cell-mediated immunity and reduce inflammatory cytokines production [113]. However, despite the potential benefits of inflammatory attenuation in the CNS, randomized controlled trials and observational studies have failed to show a definitive therapeutic benefit for the use of antirheumatic agents in the treatment of AD [117]. Further research is needed to understand the potential benefits and drawbacks, such as adverse side effects, in the use of these agents in patients suffering from AD.

Another pharmacological agent identified by our network analysis was the medication tretinoin, a vitamin A derivative. Retinoic acids, such as tretinoin, are involved in neural differentiation and patterning, and growth [118]. Tretinoin and other vitamin A derivatives have a cholinotropic effect on the CNS and restore acetylcholine levels in the brain, a mechanism of action that may prove important in the treatment of AD [119].

In contrast to the potential therapeutic effects of the chemicals mentioned above, arsenic was also identified in our chemical analysis. Arsenic is a chemical that has been documented to increase the risk of developing AD [120]. A study conducted on patients in Taiwan found that individuals living in locations with higher concentrations of arsenic, and a resultant increased urinary percent excretion of arsenic, were at a significantly higher risk of AD [120]. Proposed mechanisms of the increased risk of AD after arsenic poisoning include induction of tau hyperphosphorylation and formation of Aβ plaques as well as an increased risk of cardiovascular disease, which may contribute to vascular causes of AD as well [121]. Increased levels of arsenic in rat models were also shown to increase the number of reactive oxygen species and advanced glycosylation end products in the CNS, along with decreased serum Aβ clearance [122].

### 3.7. Limitations

Due to the method used in this study, several limitations should be considered when interpreting the data. The results were obtained from a single dataset and should be tested in independent cohorts. Another limitation may be associated with the diagnosis criteria. In the study, the patients were classified based on a clinical assessment before death and AD neuropathology using BRAAK staging at autopsy. In this study, AsymAD patients were characterized by the lack of clinical signs of AD and neuropathology at autopsy. These patients could represent a heterogeneous population. Some patients might have been preclinical and may have developed clinical symptoms if they had lived longer, whereas other patients might be resilient to cognitive decline. The sample size is another factor that could influence the results. Additional studies involving larger numbers of study participants will be needed to determine if the results reported here may be replicated.

## 4. Materials and Methods

### 4.1. Data Base Mining, SWIM Analysis to Identify Switch Genes, Switch Gene Analysis

The NCBI GEO database (https://www.ncbi.nlm.nih.gov/gds (accessed on 30 April 2020)) and ArrayExpress database (https://www.ebi.ac.uk/arrayexpress/ (accessed on 30 April 2020)) were searched for studies in which gene expression data were available from laser-captured neurons in the brain of Alzheimer’s patients. The NCBI GEO database was queried using the search terms Alzheimer’s, brain, neuron, and “Homo sapiens” (Organism) for the study types expression profiling by array and expression profiling by high-throughput sequencing. A total of 44 studies were identified, 21 were brain-specific studies, and 1 had data from laser-captured neurons that were specific for the entorhinal cortex (GSE118553). The characteristics of the participants (sex, age, disease duration) and the samples’ description (number, BRAAK score, and post-mortem delay) were previously published [11]. Table 1 summarizes their findings. Raw data from the expression arrays were imported into SWIM. The SWIM algorithm is comprised of several steps as we previously described [19,20].

### 4.2. Pathway Enrichment Analysis

Official gene symbol from the genes identified in the Switch analysis were imported into NetworkAnalyst accessed on the 6 June 2020 (https://www.networkanalyst.ca/NetworkAnalyst/faces/home.xhtml) for pathway analyses [123]. The Kyoto Encyclopedia of Genes and Genome (KEGG) pathway database was used as annotation sources [124].

### 4.3. Gene–Transcription Factor Interaction Analysis

Gene–transcription factor interactome was performed in NetworkAnalyst. Transcription factor and gene target data were derived from the Encyclopedia of DNA Elements (ENCODE) ChIP-seq data, ChIP Enrichment Analysis (ChEA), or JASPAR database [125,126,127]. ENCODE uses the BETA Minus algorithm in which only peak intensity signal <500 and the predicted regulatory potential score <1 is used. ChEA transcription factor targets database inferred from integrating literature-curated Chip-X data. JASPAR is an open-access database of curated, non-redundant transcription factor-binding profiles. A Venn diagram analysis was performed with the transcription factors identified with each database. Transcription factors were ranked according to network topology measurements, including degree and betweenness centrality.

### 4.4. Gene–miRNA Interaction Analysis

The gene–miRNA interactome was performed in NetworkAnalyst. The gene–miRNA interactome was conducted using comprehensive experimentally validated miRNA–gene interaction data collected from TarBase v.8.0 and miRTarBase v.8.0 [128,129,130]. miRNA data were ranked according to network topology measurements, such as degree and betweenness. Venn diagram analysis was then performed with the miRNA identified from the two databases that yielded results.

### 4.5. Gene–Disease Association Analysis

Gene–disease association analysis was performed in NetworkAnalyst. The literature-curated gene–disease association information was collected from the DisGeNET database, a publicly available collection of genes and variants associated with human diseases [131].

### 4.6. Gene–Chemical Analysis

Protein–chemical associated analysis was performed in NetworkAnalyst. The literature-curated gene–chemical analysis was taken from the Comparative Toxicogenomics Database, a genomic resource available to the public derived from genes and proteins of toxicologic significance to humans [132].

## 5. Conclusions

In this study, we identified key switch genes involved in the transition to AD from healthy or AsymAD. These genes revealed that chemokine signaling, platelet activation, and phospholipase D signaling pathways might be involved in the transition to clinical AD. Further, the transcription factor, PPARG, and 16 miRNAs were identified as potential switch gene regulators. Chemical–protein interaction analysis revealed that valproic acid might be a therapeutic agent that could prevent AD progression. Future studies using larger cohorts of individuals suffering from AD will be needed to assess the potential therapeutic targets related to these pathways, chemicals, transcription factors, and miRNA regulators.

## Figures and Tables

**Table 1:** Characteristics of the participants.

	Controls	AsymAD	AD
Sample number	16	28	34
Age (±SD)	71.9 (±15.6)	85.4 (±9.5)	83.9 (±9.7)
Sex (M/F)	9/7	8/20	13/21
BRAAK (±SD)	0	2.2 (±1.2)	4.9 (±1)
Disease duration (y)	0	0	11.8 (5.2)
Post-mortem delay	33.8 (17.8)	52.5 (15.9)	39.5 (21.2)

## Data Availability

Not applicable.

## Supplementary Materials

The following are available online at https://www.mdpi.com/article/10.3390/ijms22083915/s1.
