# Supplementary material for: Key Disease Mechanisms Linked to Alzheimer’s Disease in the Entorhinal Cortex
Source: Int J Mol Sci. 2021 Apr 10;22(8):3915. doi: 10.3390/ijms22083915 (PMC8069371; doi:10.3390/ijms22083915)
Supplement: Supplementary file 1 [file ijms-22-03915-s001.zip › ijms-1169227 supplemental material.v1/ijms-1169227 supplemental Figures.pdf]

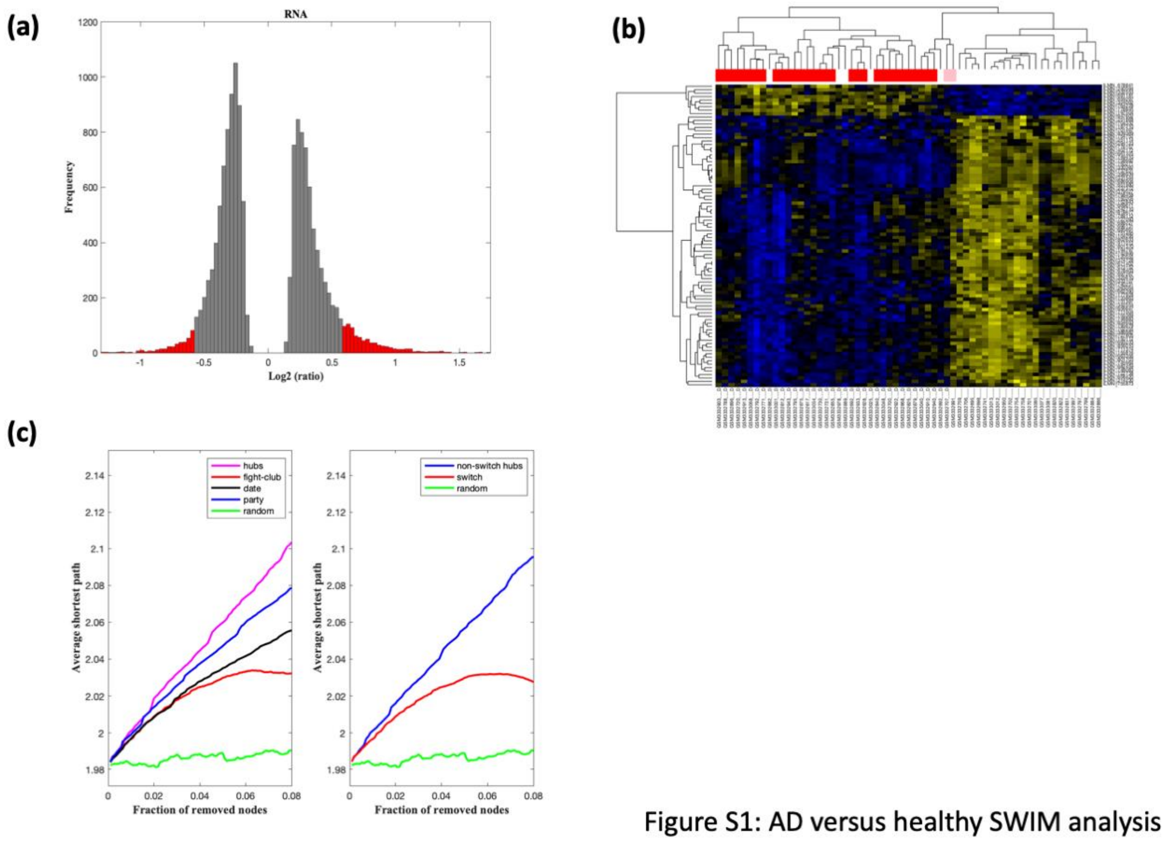

Figure S1. AD/healthy SWIM analysis. (**a**) Distribution of log2 fold change values where the red bars are selected for further analysis. (**b**) Dendrogram and heat map for switch genes. The suffix D indicates the samples from the AD cohort. The colors represent expression levels, with blue indicating downregulated and yellow indicating upregulated (**c**) Robustness of the correlation network.

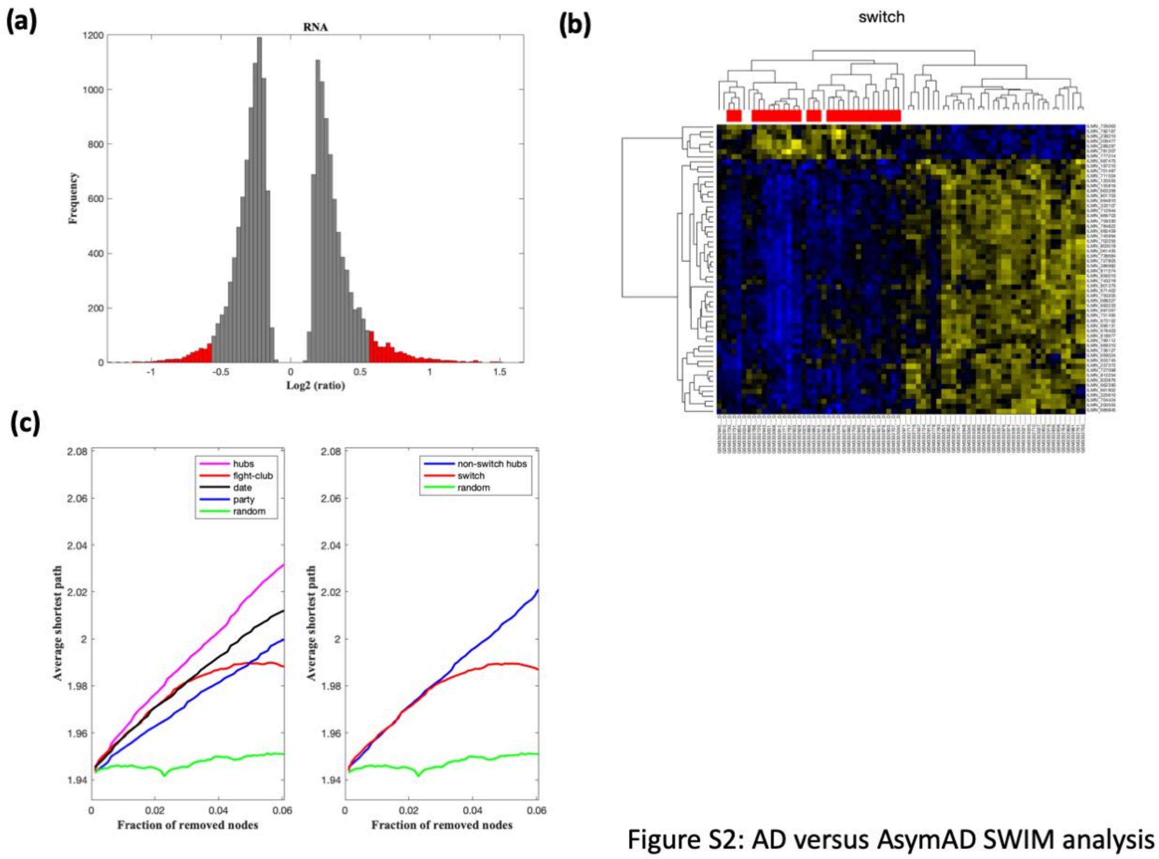

Figure S2. AD/AsymAD SWIM analysis. (**a**) Distribution of log2-fold change values, where the red bars were selected for further analysis. (**b**) Dendrogram and heat map for switch genes. The suffix D indicates the samples from the AD cohort. The colors represent expression levels, with blue indicating downregulated and yellow indicating upregulated (**c**) Robustness of the correlation network.
